# Increased level of compleasomes in cerebrospinal fluid of patients with herpes simplex encephalitis

**DOI:** 10.1007/s13365-018-0665-x

**Published:** 2018-08-09

**Authors:** Ewa Johansson, Stefan Lange, Tomas Bergström, Merna Oshalim, Ivar Lönnroth, Marie Studahl

**Affiliations:** 1 Clinical Microbiology, Sahlgrenska University Hospital, PO Box 7193, S-402 34 Gothenburg, Sweden; 2 Department of Infectious Diseases, Institute of Biomedicine, University of Gothenburg, PO Box 420, S-405 30 Gothenburg, Sweden; 3 Department of Infectious Diseases, Sahlgrenska University Hospital, Diagnosvägen 21, S-416 85 Gothenburg, Sweden

**Keywords:** Herpes simplex encephalitis, Cerebrospinal fluid, Compleasome, Antisecretory factor, Proteasome, Complement factor

## Abstract

Herpes simplex encephalitis (HSE) is a common cause of viral encephalitis (HSV-1) characterised by pronounced inflammation and elevated intracranial pressure. We have shown in a rat model that HSV-1 infection causes an interaction between complement factors and proteasomes, leading to formation of proteasome/complement complexes (compleasomes). Exposure of the proteasome regulatory subunit antisecretory factor 1 (AF1) leads to a decrease in intracranial pressure. The aim of this study was to evaluate the acute and prolonged formation of compleasomes in cerebrospinal fluid (CSF) from patients with HSE. Cerebrospinal fluid samples (*n* = 55) from 24 HSE patients were analysed for compleasome complexes. Samples from healthy controls (*n* = 23) and patient controls (*n* = 27) served as baseline information. Sandwich enzyme-linked immunosorbent assay (ELISA) for proteasomes and their complex formation with complement factor 3 or 4, and Western blot for C3 activation were performed on CSF samples. Increased compleasome formation, both presenting as an initial formation and showing exposure of subunit AF1 in the compleasomes, was found in CSF samples drawn from patients with HSE compared with samples from the control groups (*p* < 0.0005). The total protein CSF concentration was equal in all groups. The levels were higher in the acute phase compared with late in the disease course (*p* < 0.0005). Complement 3 breakdown product iC3b was detected in CSF samples of the HSE patients. The early increased formation of compleasomes in CSF suggests that this complex may be involved in host defence against HSE.

## Introduction

Herpes simplex encephalitis (HSE), most often caused by herpes simplex virus type 1 (HSV-1), induces an acute focal, necrotizing inflammation with a predilection for the frontotemporal regions of the brain. Although acyclovir treatment has been reported to reduce mortality (Raschilas et al. 2002; Sili et al. 2014; Skoldenberg et al. 1984), a large proportion of surviving patients suffer from permanent neurological sequelae, including cognitive, memory and behavioural impairments (Raschilas et al. 2002), as well as a high risk of developing subsequent epilepsy (Hjalmarsson et al. 2007). The acute intrathecal inflammatory response may be followed by long-term inflammation (Aurelius et al. 1994; Aurelius et al. 1993; Lind et al. 2017; Skoldenberg et al. 2006), which may be part of the destructive process leading to neurological damage in HSE survivors. Recently, the complement system, which is a part of the innate immune response, was found to be activated in HSE. The complement factors C3a, C3b, C5 and C5a were increased in the cerebrospinal fluid (CSF) from HSE patients compared with healthy controls, and C3a and C5a concentrations remained increased at long-term follow-up (Eriksson et al. 2016). The innate immune response aims to suppress viral replication and cell death and also enhances adaptive immunity (van Beek et al. 2003; Veerhuis et al. 2011). In addition, the proteasome system has emerged as regulator of innate immune responses and different types of proteasomes have been detected in the CNS (Ramachandran and Margolis 2017). In CSF, extracellular proteasomes have been suggested to play a role in neurodegenerative inflammatory diseases of the CNS (Mueller et al. 2012). One component of the regulatory part of the proteasome is antisecretory factor 1 (AF1, also named S5a/rpn10/PSMD4) (Tomko Jr and Hochstrasser 2013). The 43-kD ubiquitously expressed AF1 protein has been shown to exert a potent antisecretory and anti-inflammatory effect (Davidson and Hickey 2004; Johansson et al. 1995; Lange and Lonnroth 2001). The 16-amino acid-long sequence 36–51 has been designated AF-16 and on account of its stability and potency is used in experimental work (Johansson et al. 1997; Matson Dzebo et al. 2014; Nicolas and Lievin-Le Moal 2015). Intranasally administered AF-16 in rats experimentally infected with HSV-1 has been demonstrated to abolish sickness and death during time course of infection (Jennische et al. 2008), most likely accomplished by an AF-16-mediated absence of increased intracranial pressure (ICP) ubiquitously found in HSE-affected rats. Decreasing of a high ICP in infected rats has also been achieved by a diet-induced rise in endogenous AF1 (Johansson et al. 2013). The role of a high ICP is scarcely investigated in human HSE, but since a high ICP leads to decreased consciousness which is associated to poor prognosis in HSE (Singh et al. 2016), a high ICP might be a bad prognostic factor (Jouan et al. 2015; Barnett et al. 1988) and a target for therapy.

We have previously described that during the initial phase of infection, a complex is formed between circulating proteasomes and complement factors, which we have named the “compleasome” (Lonnroth et al. 2016). During formation of compleasomes, the C3 complement factor is split into C3c and C3d. The proteolysis of C3 into C3c has been described as deactivation of complement activity (Gros et al. 2008). This split changes the proteasome conformation, resulting in an exposure of previously hidden antisecretory epitopes. Thus, rats infected with HSV-1 for experimental encephalitis studies were shown to have induced compleasomes in CSF (Lange et al. 2016). However, no hydrolyses of C3 or exposure of AF occurred in that complex. This was probably because, for ethical reasons, the rats were sacrificed before clinical symptoms developed (Lange et al. 2016). Since AF-16 abolished HSE in HSV-1 infected rats, it is of interest to study the benefit formation of AF compleasomes in human CSF during HSV-1 infection.

The aim of the present study was to investigate the formation of compleasomes in human CSF in response to HSE, and to study this response over time.

## Materials and methods

### Antibodies, proteasome and complement proteins

Immunoglobulin (Ig)M monoclonal antibody (mAb) against AF1/RPN10 was produced as previously described (Johansson et al. 2009). Monoclonal mouse IgG antibodies against proteasome 20Sα6 and the proteasome 26S and 20S proteins were obtained from Enzo Life Sciences, Inc. (www.enzolifesciences.com). Polyclonal antibody (pAb) against proteasome 19S subunit was produced as previously described (Lonnroth et al. 2016), and pAb against C3 and C4 was obtained from Dako, Glostrup, Denmark. Secondary antibodies, alkaline phosphatase (AP)-conjugated goat anti-rabbit IgG, and goat anti-mouse IgM were obtained from Jackson ImmunoResearch Europe Ltd., Västra Frölunda, Sweden. The complement factor C3 (Sigma-Aldrich, Sigma-Aldrich Sweden AB, Stockholm, Sweden) and the split products C3b and iC3b were obtained from Calbiochem, Merck Chemicals and life Science AB, Solna, Sweden.

### Study population and samples

#### Patients and samples

Patients with HSE admitted to the Department of Infectious Diseases, Sahlgrenska University Hospital, Gothenburg, Sweden, between 1995 and 2014, constituted the study population. They had clinical signs of encephalitis with fever, disorientation, altered consciousness, paresis, seizures, and/or dysphasia, as well as magnetic resonance (MR) or computed tomography (CT) findings compatible with herpes encephalitis. The diagnosis was confirmed on admission to hospital by detection of HSV-1 DNA using quantitative in-house TaqMan PCR methods (Namvar et al. 2005). The CSF samples were centrifuged at 1500 rpm for 5 min to remove cells, aliquoted and stored at −70 °C. Inclusion criteria in the study were a confirmed diagnosis of HSE (*n* = 24) and sufficient lumbar CSF (55 samples). We defined acute infection (“early samples”) as days 0–10 after onset of neurological symptoms. Samples after day 10 were defined as continued inflammation/late infection (“late samples”). For included HSE patients, number of days after onset of neurological symptoms was noted and the medical records were examined. Treatment data were retrieved, and outcome, measured after 6 months, was reported according to the Glasgow Outcome Scale (GOS) (Jennett and Bond 1975), a scale of 1–5, where 1 indicates death and 5 indicates low, or no, disability where the patients can return to the life they had before the infection. Fourteen out of 22 patients (63.6%) had moderate disability or good recovery (GOS 4–5), and eight (36.4%) had worse outcome (GOS 1–3) at 6 months.

#### Healthy controls and patient controls and samples

Decoded samples of CSF were obtained from healthy controls (HC; *n* = 23) and patient controls (PC; *n* = 27). The healthy controls were recruited as next of kin to patients suffering from amyotrophic lateral sclerosis (*n* = 23), and samples were collected at the Department of Neurology, Sahlgrenska University Hospital, Gothenburg, Sweden. The healthy controls had no history or symptoms of signs of psychiatric, neurological, malignant, or systemic disorders. The patient controls were subjects who were seeking medical care and were lumbar punctured because of headache and/or psychoneurotic symptoms and in whom CNS infection initially was suspected, but later excluded. Exclusion of CNS infection was based on a non-pathological neurological status and lack of pleocytosis in the CSF. Analysis of patient control samples revealed normal CSF cell count and, for most, normal CSF albumin concentrations. Characteristics of the clinical sample collection are presented in Table 1.

**Table 1:** Demographics of herpes simplex encephalitis (HSE) patients, healthy controls and patient controls

	HSE patients (*n* = 24)	Healthy controls (*n* = 23)	Patient controls (*n* = 27)
Male/female ratio	14:10	14:9	18:9
Mean age in years (range)	57 (27–89)	56 (27–73)	47 (28–69)
Median number of days after onset of neurological symptoms for the first sample (interquartile range)	18 (9–46)	n.a.	n.a.
GOS^a^ score (*n* = 22)	Number of patients	n.a.	n.a.
1	3		
2	–		
3	5		
4	3		
5	11		
Number of samples collected at acute infection or prolonged inflammation/late infection			
0–10 days after onset of symptoms	12 (*n* = 8)		
11–523 days after onset of symptoms	42 (*n* = 22)		
Unknown	1 (*n* = 1)		
Number of samples tested per individual			
1 sample	11	23	27
2 samples	5		
3 samples	5		
4 samples	1		
5 samples	1		
10 samples	1		

^a^Glasgow Outcome Scale (Jennett and Bond 1975), where 1 = death, 2 = persistent vegetative state, 3 = severe disability, 4 = moderate disability, and 5 = good recovery

*n.a.* not applicable

The ethical committee of the University of Gothenburg approved this study (Dnr. 664-13).

### Compleasome enzyme-linked immunosorbent assay

A sandwich enzyme-linked immunosorbent assay (ELISA) for detection of compleasomes (proteasome/complement complexes) in CSF was performed as previously described (Lange et al. 2016; Lonnroth et al. 2016). Two variants of the compleasome ELISA were performed to distinguish the initial formation of compleasome and the exposition of specific proteasome subunit AF1 in the compleasome. Two mAbs against proteasome protein subunits 20Sα6, or AF1, with phosphate-buffered saline (PBS) as control, were coated on a Costar® 96-well, half-area, high-binding polystyrene plate (Fisher Scientific, GTF AB, Gothenburg, Sweden) overnight (dilution 1:2000, except for mAb AF1 which was diluted 1:400). After blocking with 0.2% bovine serum albumin (BSA) at 37 °C for 45 min, CSF samples from HSE patients (*n* = 23–24), PCs (*n* = 25 or 27), and HCs (*n* = 23) were titrated in PBS with 0.2% BSA and 0.05% Tween 20, and shaken for 2 h. A pAb (anti-C3 or anti-C4) at 1:2000 dilution was used as the detecting antibody. An anti-rabbit-AP secondary antibody was applied after 1 h of incubation, and AP substrate was added after a further hour. Absorbance was read at 405 nm in a photometer, and the difference between antibody-coated samples and controls (PBS) was estimated.

### Sodium dodecyl sulphate-polyacrylamide gel electrophoresis and Western blot

The content of C3 products was determined in CSF samples collected from healthy individuals (*n* = 2) and from patients with HSE (*n* = 3) by sodium dodecyl sulphate (SDS)-polyacrylamide gel electrophoresis (SDS-PAGE) followed by immunoblotting onto nitrocellulose membrane. The membrane was blocked with 1% BSA in PBS at 4°C for 16 h. The primary polyclonal anti-C3 (diluted 1:25,000) was then added to the membranes and incubated for 1.5 h. The blot was developed with AP-conjugated goat anti-rabbit IgG followed by 5-bromo-4-chloro-3-indolyl phosphate and 4-nitro blue tetrazolium (Roche Diagnostics, Mannheim, Germany).

### C3c detection

C3c was detected and quantified in CSF from HSE patients (ten early samples and 18 late samples) and patient controls (13 samples) using a C3c assay kit (human C3c ELISA kit HK368, Hycult Biotech/Nordic Biosite, Stockholm, Sweden), following the manufacturer’s instructions. The C3c concentration of selected CFS samples, which were run parallel with standards, was determined from the standard curve.

### Proteasome enzyme-linked immunosorbent assay

Sandwich ELISA detection of 26S proteasomes in CSF from HSE patients (15 samples from 12 patients, collected on days 5–523 after onset of symptoms) and healthy controls (*n* = 23) was performed as previously described (Lonnroth et al. 2016). The capturing mAb against 20Sα6 diluted 1:1000 was coated on a Nunc-Immuno 96-well MaxiSorp (Sigma-Aldrich), and the polyclonal anti-19S proteasome subunit, diluted 1:400, was used as detecting antibody. 26S proteasome was used as reference and rabbit pre-immune serum as control. The same protocol was used as previously described for the compleasome ELISA.

### Protein determination

The protein content in CSF from patients with HSE (*n* = 9), PCs (*n* = 10) and HCs (*n* = 10) was determined with the Pierce BCA protein assay kit (Thermo Scientific GTF AB, Gothenburg, Sweden) reading absorbance at 562 nm using a NanoDrop photometer (Saveen Werner, Malmö, Sweden).

### Statistics

GraphPad Prism version 7 (GraphPad Software Inc., La Jolla, CA, USA) was used for statistical analysis and graph construction. Data are presented as mean ± standard deviation (SD). Statistical significance of difference (*p* value) for two means was assessed using an unpaired Student’s *t* test; for three means, analysis of variance (ANOVA) was used and *p* < 0.05 was considered significant.

## Results

### Induction of compleasome complexes by herpes simplex virus type 1

**Fig. 1:**
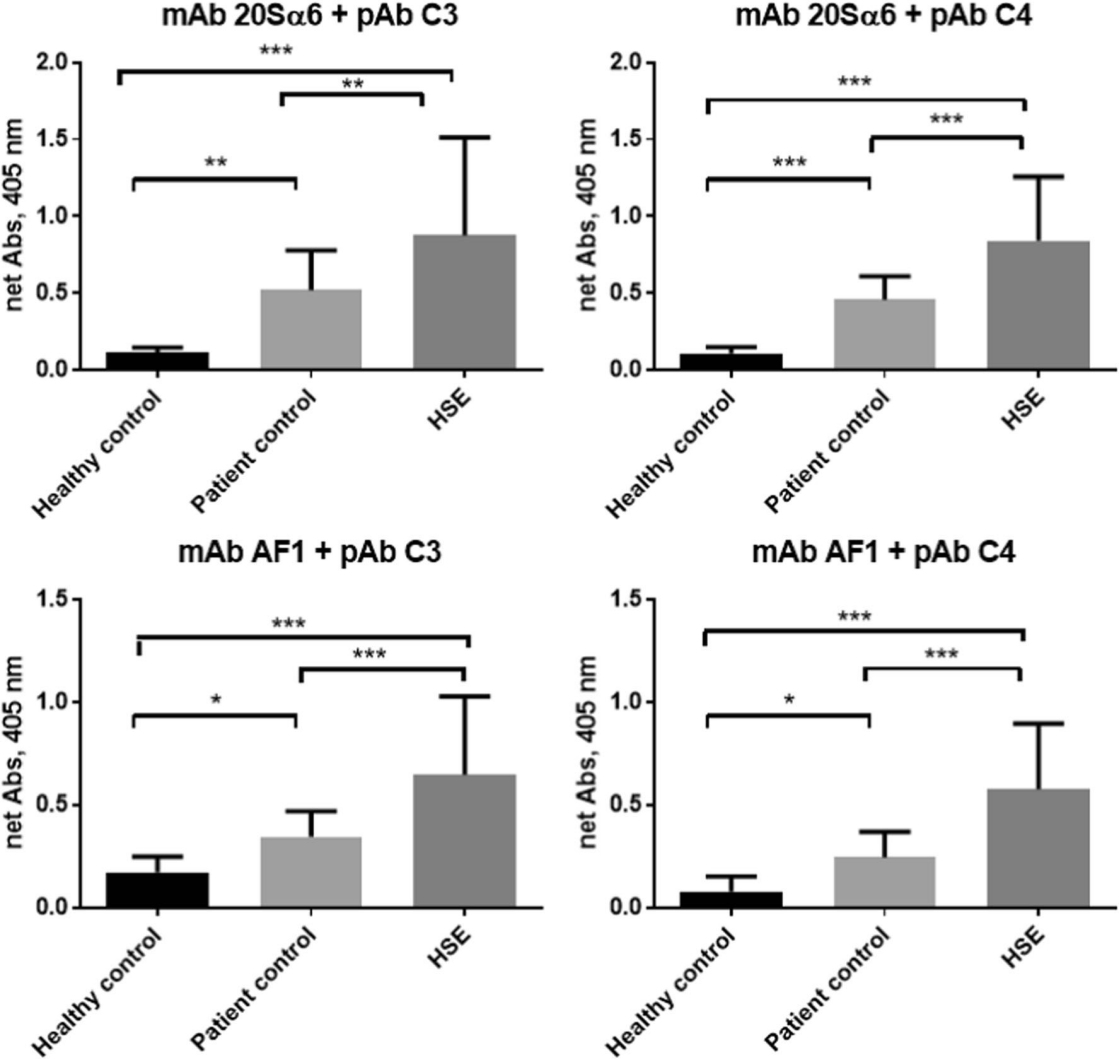
Detection of compleasome complexes in cerebrospinal fluid (CSF) taken from healthy controls (HCs), patient controls (PCs) and herpes simplex encephalitis (HSE) patients using sandwich enzyme-linked immunosorbent assay (ELISA). The figure presents results from four combinations of antibodies using monoclonal antibodies (mAb) against proteasome subunit 20Sα6 or AF1 as catching antibodies and polyclonal antibodies (pAb) against complement factors 3 and 4 (C3 and C4) as detecting antibodies. The patients with HSE (*n* = 23 or 24) had significantly higher values compared with the HCs (*n* = 23) and also with the PCs (*n* = 25 or 27) tested with all four combinations of compleasome antibodies. Data are expressed as mean absorbance values, buffer blank corrected, ± standard deviation (SD). Significant differences between the samples are indicated by *p < 0.05, **p < 0.005 and ***p < 0.0005

Figure 1 presents four combinations of antibodies using mAbs as catching antibodies against the proteasome subunit 20Sα6, or AF1 together with pAbs for detection of antibodies against complement factors 3 or 4 (C3 and C4). The levels were significantly higher in the HSE group compared with both control groups analysed with all four combinations of antibodies (*p* < 0.0005). The compleasome levels found after using catching antibody against proteasome subunit 20Sα6 represent the intact compleasome in CSF. Use of mAb AF1 as catching antibody in the sandwich ELISA indicated increased exposure of the specific subunit AF1 in these samples obtained from HSE patients. The levels detected in samples collected from PCs were also somewhat increased compared with CSF obtained from HCs. However, the induction of the compleasomes exposing AF1 was lower compared with formation of initial compleasome (Fig. 1). The proteasomes in CSF were bound to C3 and C4 in a similar ratio.

### Compleasome levels in acute and late infection

**Fig. 2:**
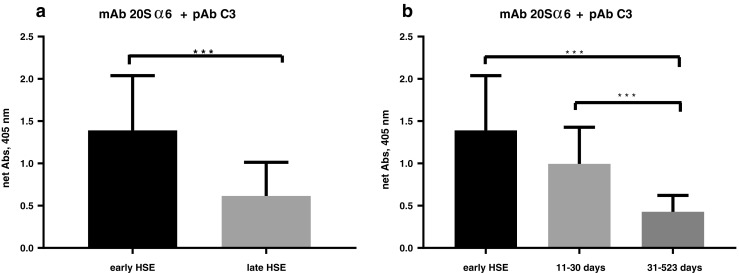
Cerebrospinal fluid (CSF) levels of compleasome in herpes simplex encephalitis (HSE) patients, separated into early, acute (12 samples from 8 patients) and late infection (42 samples from 22 patients). The complex was measured with sandwich enzyme-linked immunosorbent assay (ELISA) using monoclonal antibody (mAb) against proteasome subunit 20Sα6 as catching antibody and polyclonal antibody (pAb) against complement factor 3 (C3) as detecting antibody. **a** Samples of CSF collected early in the infection at the acute phase had significantly higher levels of compleasome compared with samples collected more than 10 days after onset of symptoms. **b** Data from late infection were further separated into 11–30 days and 31–523 days after onset of symptoms. The level of compleasome had significantly decreased in the CSF samples collected after the first 30 days. Data are expressed as mean ± standard deviation (SD). Significant differences (*p* < 0.0005) between the samples are indicated by ***

The formation of compleasomes after HSV-1 infection in early and late CSF samples was examined by sandwich ELISA. As shown in Fig. 2a, the 12 samples collected from eight patients in the acute phase showed a highly significant increase in the compleasome complex compared with the samples collected more than 10 days after onset of symptoms (42 samples from 22 patients) (*p* < 0.0005). The late samples were then divided into two subgroups, collected at days 11–30 (14 samples) and between 31 and 523 days (28 samples) after onset of symptoms. Levels were compared with the samples from early HSE (Fig. 2b). The level of compleasome seems to sequentially drop over time after the initial HSV-1 infection. The middle samples showed a tendency of decreasing levels (*p* = 0.0769), compared with the highly significant decrease in the compleasome complex detected in the samples collected very late (*p* < 0.0005).

We compared patients’ outcome according to GOS and the level of compleasome in CSF during acute HSV-1 infection where data were available (*n* = 7). A tendency (*p* = 0.0541) of higher levels of compleasome was seen in samples taken from patients (*n* = 4) with good recovery (GOS 5) compared with patients (*n* = 3) with worse outcome (GOS 1–4).

### Compleasome levels in follow-up samples

**Fig. 3:**
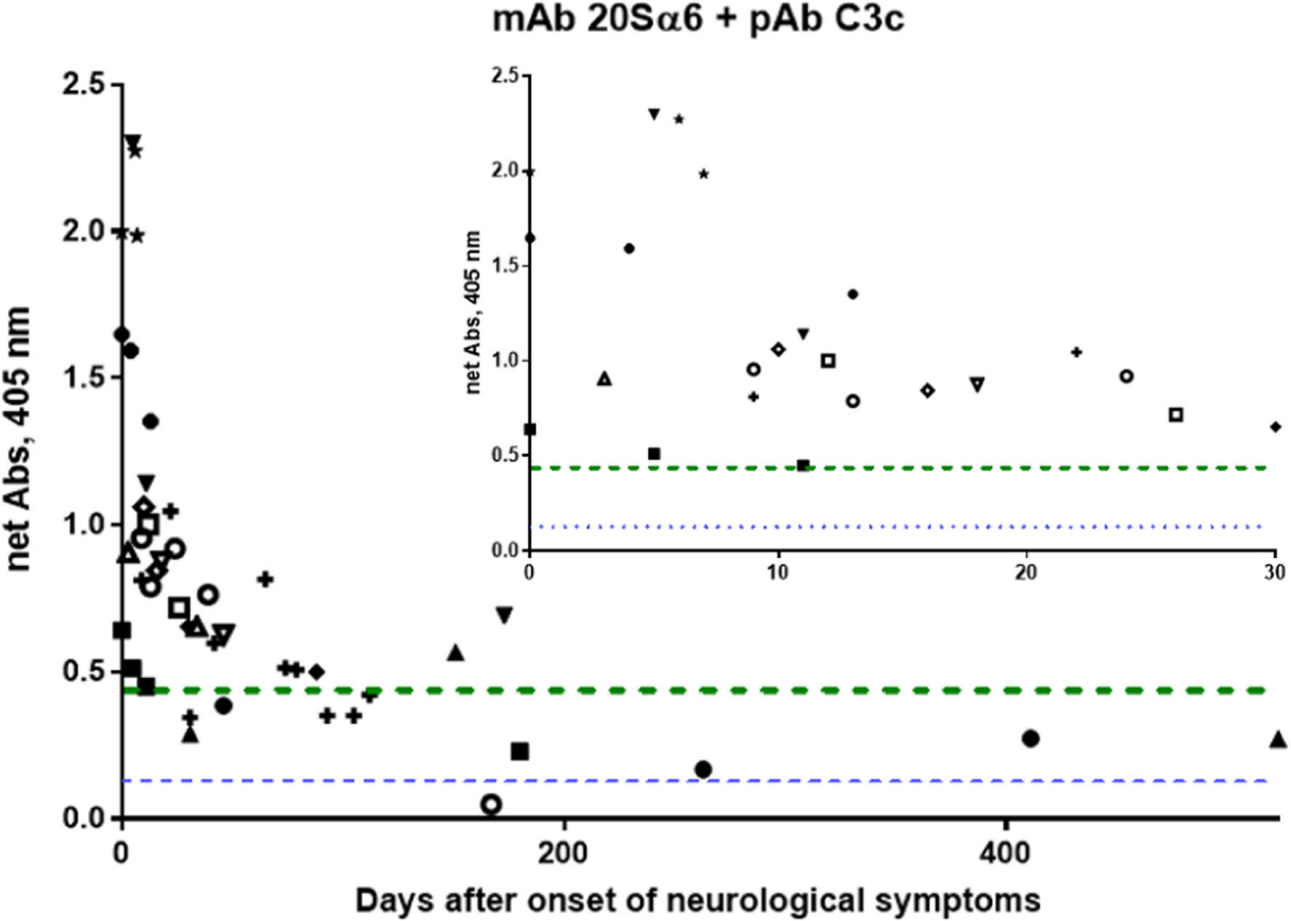
Levels of compleasome complex in cerebrospinal fluid (CSF) samples from herpes simplex encephalitis (HSE) patients over time (*n* = 13). The complex was measured with sandwich enzyme-linked immunosorbent assay (ELISA) using monoclonal antibody (mAb) against proteasome subunit 20Sα6 and polyclonal antibody (pAb) against complement factor 3 (C3) and is presented in relation to day of onset of neurological symptoms. Between two and ten samples of individual patients from different time points (collected 0–523 days after symptom onset) are presented. The inset shows a detailed graph of the CSF samples collected after 0–30 days. Median levels of compleasome complex in CSF of healthy controls (HCs) (*n* = 23) are indicated with a dotted line and of patient controls (PCs) (*n* = 27) with the dashed line

We also investigated formation over time of compleasome in CSF from individual HSE patients with more than one sample available (between two and ten samples, collected at between day 0 and day 523, from 13 patients) by constructing a graph. The compleasome levels in the follow-up samples showed a distinct pattern whereby early formation of compleasomes was followed by a decline over time (Fig. 3). A detailed graph of the compleasome formation for the first 30 days of HSE disease clearly shows the high levels of compleasome during acute infection.

### Complement factor 3 degradation in cerebrospinal fluid

**Fig. 4:**
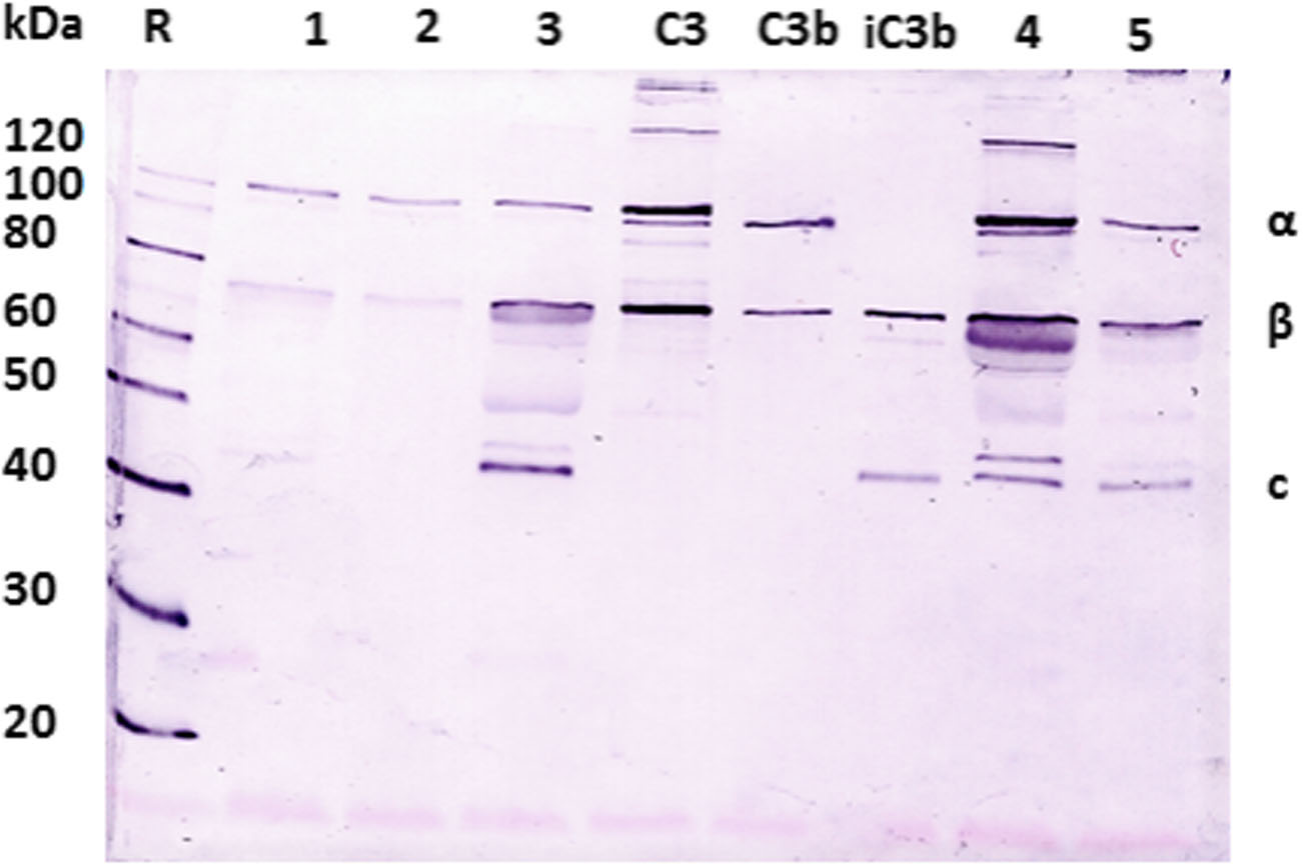
Western blot analysis of cerebrospinal fluid (CSF) using antibody against complement factor 3 (C3). Cerebrospinal fluid collected from healthy individuals 1 and 2 shows antibody reaction to C3b while the CSF from HSE patients 3, 4 and 5 showed the degraded form of C3b and iC3b. The sample from patient 4 was taken during acute HSE infection on day 7 after onset of symptoms, showing a stronger antibody reaction compared with the sample from patient 5, taken on day 173, and patient 3, taken at day 16 after onset of symptoms. The split products of C3 into α, β and c chain fragments are indicated to the right. The molecular weight standard (R) is applied to the left

Since C3 degradation is involved in the formation of compleasome, the level of C3 breakdown products in CSF from HSE patients was investigated by Western blot using C3 antibody. As Fig. 4 demonstrates, the C3 cleavage fragment iC3b was detected in HSE patients (*n* = 3) while degradation products were absent in HC samples (*n* = 2). The reaction was strongest in samples collected during early infection (day 7), compared with a weaker signal in samples taken on day 16 and day 173 after onset of symptoms.

We quantified the level of C3c in CSF in samples collected early (*n* = 10) and late (*n* = 18) in HSE, together with samples taken from PCs (*n* = 13) using the C3c assay kit. Samples collected early in the infection at acute phase had significantly higher levels of specific C3c compared with samples collected more than 10 days after onset of symptoms (*p* = 0.0036) and samples from PCs (*p* = 0.0033) (Table 2).

**Table 2:** Level of specific complement factor 3c (C3c) in cerebrospinal fluid (CSF) collected from herpes simplex encephalitis (HSE) patients at acute infection and at prolonged inflammation/late infection, and from patient controls (PCs)

	C3c level		
	*Low* < 60 ng/ml, *n* (%)	*Moderate* 60–120 ng/ml, *n* (%)	*High* > 120 ng/ml, *n* (%)
Patient controls (*n* = 13)	13 (100)	0	0
HSE patients (0–10 days after onset of symptoms) (*n* = 10)	5 (50)	3 (30)	2 (20)
HSE patients (14–294 days after onset of symptoms) (*n* = 18)	17 (94.4)	1^a^ (5.5)	0

^a^Sample taken at day 14 after onset of symptoms

There was no significant difference in protein concentration in CSF in the PCs and HCs compared with the HSE patients, the respective levels being 0.72 ± 0.18 mg/ml, 0.71 ± 0.17 mg/ml and 0.86 ± 0.36 mg/ml (*p* = 0.339 and *p* = 0.280). This finding suggests no leakage of proteins from blood to CSF in the HSE patients. Therefore, low levels of proteasomes were detected in CSF collected from HCs (1 ± 1 ng/ml), while the HSE samples contained a high level of proteasomes (50 ± 13 ng/ml).

## Discussion

In the present work, we demonstrate an increased CSF compleasome concentration in response to HSE, especially during the acute phase of the disease. The early formation of compleasomes was followed by declining compleasome levels over time. This kind of kinetic response suggests an important role for this specific protein complex in the intrathecal innate host defence against HSE infection.

This early formation of compleasomes in human CSF is similar to the one we found in our previous investigation of HSV-1 rat encephalitis. In that study, we demonstrated an early response of proteasome subunit 20Sα6 compleasomes in CSF, which did not respond to AF1 antibodies (Lange et al. 2016). In the rat model, the response after the acute phase was not investigated, since the rat encephalitis was lethal. By contrast, the present study of human CSF revealed a significant AF1 compleasome response during or after the HSV-1 acute phase of the disease. This result suggests that a total conformational change had occurred in the compleasome as a result of the HSV-1 infection. Our previous in vitro experiments suggest that AF1-reactive compleasomes are formed in a later phase of the disease, in close correlation to a C3 complement degradation (Lonnroth et al. 2016). This theory was confirmed in the present study. Thus, the HSV-1 infection induced a considerably stronger compleasome formation in the acute phase compared with a later stage of the disease. This fast reactivity against the infection can be interpreted as a most essential part of the innate immune response with the purpose of counteracting infections in the brain.

The compleasome levels were also enhanced in CSF collected from PCs compared with HCs. The PCs had undergone lumbar puncture for a suspected clinical CNS infection. However, CNS infection was ruled out because of lack of CSF pleocytosis. These PCs all had headache and other unspecific symptoms, which might have been elicited by an infection of unknown origin, without specific involvement of the meninges or the neuronal brain tissue, and these symptoms with unknown aetiology had induced compleasome formation. However, the compleasome formation was lower in the PC group compared with the HSE group.

The formation of compleasomes declined over time. Ten days after the onset of neurological symptoms was arbitrarily chosen, but the time point generally corresponds well to the disappearance of acute symptoms. Levels of HSV DNA in CSF usually start declining at 10–21 days (Schloss et al. 2009). There is probably a continuous development of the immunological processes in the brain, and the time points at which lumbar puncture was performed may therefore reflect different stages of the disease. It is well known that prodromal symptoms, sometimes lasting for up to a week, are present in approximately half of HSE cases, while the onset of disease is more abrupt in others (Kennedy et al. 1988). We can, however, conclude that compleasome formation significantly and continuously decreases after the initial month of HSE disease.

The acute CSF compleasome levels were higher in patients with good recovery (GOS 5) compared with patients with worse clinical outcome (GOS 1–4). This suggests that a rapid and strong formation of the innate compleasome complex may be important in the control of brain HSV-1 infection. The retrospective design of this study and analysis of frozen CSF samples imply limitations, i.e. lack of standardised timing of lumbar punctures and selected bias when using stored samples. However, the acute and late CSF samples in this study were from patients with both good and bad outcome, since the patients had previously participated in studies with long-term follow-up. The clinical data with outcome measurements (GOS) after 6 months were accurately documented in the patient’s records. Intrathecal complement activity has been examined in other CNS infections such as cryptococcal meningitis, although no test of correlation with the clinical outcome was performed (Shen et al. 2017). In a previous study, the pro-inflammatory anaphylatoxins C3a and C5a were significantly increased in CSF samples from HSE patients in the acute phase. The concentrations of C3a, C3b, C5 and C5a were, however, also increased in the late phase of infection. Thus, complement activity contributes to inflammation during both the acute and the later phase of HSV-1 infection (Eriksson et al. 2016). An earlier reduction in C3a activity was associated with better clinical outcome, measured by GOS, after 6 months (Eriksson et al. 2016). These results suggest that some inflammatory responses may contribute to CNS protection, while other components of the inflammatory response may be associated with brain tissue damage.

Suppression of the immune responses is essential for reducing incidence of late detrimental intrathecal inflammation. Therefore, the kinetics of the different parts of the immune responses in the brain must be defined and separated. When this has been accomplished, the most appropriate time point for immunosuppressive treatment can be determined. In mice, one study showed that early administration of corticosteroids was not at all beneficial compared with administration 3 days later. This finding indicates that selecting the opportune time point for immunosuppressive treatment during the disease course is essential when aiming to regulate different immune responses to HSE (Sergerie et al. 2007).

The described effects of AF1 and of its complex relation to complement factors suggest that AF compleasomes are part of the innate immune system. The N-terminal residues 5–188 of the sequence coding for AF1 contain a motif related to the von Willebrand factor A (vWA) domain. It has been described elsewhere that proteins incorporating von Willebrand factor (vWF) motifs participate in several biological events via interaction with other proteins (Edwards and Perkins 1996). Complement factor 3 plays a central role in the complement system via conformational changes to activated fragments, resulting in C3c, the physiological down-regulation product of C3 (Gros et al. 2008). Thus, vWF acts as a cofactor for factor I-mediated cleavage of C3b to inactive iC3b, thereby shutting down complement activation (Feng et al. 2015). Noone et al. suggest that vWF has a protective effect on endothelial cells and complement-mediated injury by functioning as a complement regulator (Noone et al. 2016).

## Conclusion

The present results demonstrate that AF compleasomes are formed in CSF during HSV-1 infection. The levels of compleasome in CSF were correlated to the number of days after onset of symptoms; at acute phase of infection, the HSE patients demonstrated higher levels of CSF compleasomes compared with the later stage of infection. The rapid formation of compleasomes in response to the HSV-1 infection suggests an important role in the host defence immune system of this specific complex.
